# Reflex-Neuroses and Nose Diseases: Contribution to Nasal Surgery

**Published:** 1883-03-20

**Authors:** Wilhelm Hauck

**Affiliations:** Privat-docent in the Freiburg University


### Southern Medical Record:

EDITORS:

T. S. POWELL, M.D. W. T. GOLDSMITH, M.D. R. C. WORD, M.D.

*R. C. WORD, M.D., Managing Editor.*

☞All Communications and Letters on Business connected with the RECORD must be addressed to the Managing Editor.

VOL. XIII. ATLANTA, GA., MARCH 20, 1883. No. 3.

### ORIGINAL AND SELECTED ARTICLES.

### REFLEX-NEUROSES AND NOSE DISEASES: CONTRIBUTION TO NASSAL SURGERY.

BY DR. WILHELM HAUCK,

Privat-docent in the Freiburg University. Translated by J. Edwin Micheal, M. D.

MUSCÆ VOLITANTES AND SUPRA-ORBITAL NEURALGIA.

Case 4 is that of a patient—otherwise perfectly healthy—who, after twenty-two years' military service,on purely personal grounds left the army and sought civic employment. He was certainly not of a "neurophatic disposition." Patient was never really sick; his only complaint was attacks of coryza,from which he very frequently suffered. During the last few years he had observed that the attacks of coryza occurred less frequently, but on the other hand a new symptom gradually made its appearance; a suddenly beginning glimmering before the eyes in the form of brilliant zig-zag lines and curves, which remained for hours, and was accompanied by typical supra-orbital neuralgia. The attacks were growing more frequent and more severe, so that they interfered very materially with his business. With much trouble I discovered in the otherwise perfectly normal nasal cavity, on the left inferior turbinated bone, a small patch of deep red granulations, very sensitive when touched by the sound. These I cauterised with the galvano-caustic at a single sitting. Since then his attacks ceased and have not again returned.

In this case there are several points of interest: Note the dependence of the bilateral attacks on a unilateral nose affection.

Bearing upon the connection between the symptoms described above and the nose trouble, I have been able to find only one case recorded in which there was presumably a similar connection, in which, however, no corresponding therapeutic measures were taken.

Long before I had busied myself with these conditions I was led by a sort of *argumentum ad hominem* to assume this inter dependence. In my own case there occurs a short attack of *muscæ volitantes* in the form of the well-known "fortification line" as the precursor of a cold in the head, and the attack generally ends with repeated sneezing.

These circumstances are, however, as I believe, susceptible of a much broader interpretation. It is not unthinkable that we occasionally see cases of amblyopia and amaurosis whose origin can be found in reflex nasal irritation. The literature of the day gives this idea analogical support. Very striking are the reflexes brought about in sensitive persons by inhalations of powdered ipecacuanha; we sometimes see bronchial asthma, sometimes also amblyopia and transient amaurosis. (Thamhayn, Dyce Duckworth). Observations are also tolerably numerous in which pathological irritation of other branches of the trigeminus have produced reflex amaurosis, which could only be cured after the causative irritation had been removed. (Mackensie, Hutchinson, Campbell and others.)

A word more in regard to the accompanying supra-orbital pain. It is interesting to note that in this case a typical neuralgia with characteristic symptoms—the point of greatest pain at the supra-orbital foramen was very decided—was cured by operative removal of a nasal trouble. As it is well-known this neuralgia has been associated by several authors (Mandach, Seeligmuller, and others) with catarrhal inflammation of the frontal sinuses. I do not see the necessity of this hypothesis and believe that many things mentioned by these authors can be better explained by the assumption of the reflex nature of the trouble as a result of disease of the nasal cavity. But I cheerfully admit that the question could only have been definitely settled by a careful rhinoscopic examination of the posterior nares. The greater, therefore, is my regret that the writers mentioned, in the richness of their material—Mandach could have compared 82 cases—did not communicate their results from this point of view. Zuckenkandl has examined the sinuses of the skull in 150 cases and found that the antrum of Highmore is most frequently affected with catarrh, after that the spenoidal sinuses and least frequent of all the frontal sinuses.

### CILIARY NEURALGIA.

Patient No. 5 had been operated on for nasal polypus not less than twelve times, the operation being always done with forceps. The development of each relapse was announced, long before notable stenosis had been produced by attacks of severe pain, which the patient localised partly in the lower eyelid but chiefly in the eyeball on both sides. I extirpated the polypi, whose presence in no wise interfered with respiration, with the galvano-caustic snare, and besides that thoroughly cauterized their bases. Immediately after the operation the patient had one of his former attacks lasting several minutes, but he has been entirely free from them since.

### PAIN IN THE EYELIDS.

The sixth patient suffered at first with very frequent attacks of coryza, but later during the intervals of the attacks with pains in the angle of the right eye, radiating into the upper and lower lid. The diagnosis was here very easy. There was a considerable thickening of the mucous membrane over the right lower turbinated bone, which had nevertheless produced no appreciable stenosis of that side of the nares. After a few cauterisations, which on account of the incredible terror of the patient, could not be thoroughly done, the subcutaneous pains completely vanished and, a fact of great interest, the attacks of coryza became much less frequent.

### CEPHALALGIA.

Under this head I might include two cases in which the patients complained of persistent pain and sense of pressure over the front part of the head—these troubles not assuming the character of hemicrania or neuralgia.

In the first case (7) the patient, a gentleman in the thirties, in addition to the before-mentioned symptoms, complained of attacks of severe pain, which, beginning at the upper part of the nose, radiated backward toward the base of the skull (probably following the course of nervus spinosus trigemini). In examining the nose from the front I discovered a decided thickening of the mucosa over the right middle turbinated bone. But in attempting to examine by way of the mouth, I met with an apparently insuperable irritability. The patient vomited and strangled before the instrument had touched any of the parts; so I satisfied myself as best I could with what I had gained by the anterior examination, cauterized the mucous thickening and had the satisfaction to see the frontal pain and sense of pressure vanish. The peculiar pain in the roof of the pharynx, however, remained as severe as ever. As the last-named complaint after several weeks seemed to get no better, but on the contrary to grow worse, I again undertook to make a posterior examination, and after several vain attempts, which about used up all my patience, I at last succeeded. I discovered in the left cavity a polypus as large as a hazlenut. The patient by this time was so well trained that I undertook the extirpation of the polypus through the mouth with the galvano-caustic loop with the result of immediate disappearance of all the subjective symptoms. In a few months there was a relapse. After the second removal I thouroughly cauterized the base. Since that time no returning of subjective or objective symptoms.

I cannot abstain from calling attention to this case as an illustration that the most exquisite sensitiveness of the faucial parts cannot present an insuperable obstacle to posterior rhinoscopy. One should not allow himself to be discouraged by repeated failure as was formerly too often the case with myself.

The following case (8) deserves consideration, because it touches upon the question as to whether nasal affections may not be worthy study from the psychiatric point of view. This patient had for many years suffered from numberless attacks of coryza. Gradually the frontal pain and head pressure, which accompanied the attacks, began to persist during the intervals, thereby greatly interfering with the mental work to which the patient, on account of his calling, exclusively devoted himself. His memory became poor, so that, (as he admitted to me only long after his cure), he suffered with the fixed idea that he was afflicted with a brain disease, became morose and looked to the future with the darkest forebodings. Many of the physicians whom he consulted had prescribed tonics and many other remedies but had left the nose trouble to take care of itself. Upon examination with a short speculum the nostrils on both sides appeared to be free. Upon dilatation, however, and the use of Markusoruski's long armed speculum, I discovered hanging down in the middle meatus on both sides a mass of polypi of all sizes. I removed them at several sittings with loop, forceps and galvano-caustic. There followed cure of all the subjective symptoms, return of the memory and such a gay and cheerful view of life that the patient felt himself completely transformed.

### PAIN IN ONE SIDE OF THE FACE.

I report the following case (9) with reserve: The patient, a middle-aged married woman, related that she had suffered for years with very severe pain in the left side of the face. The attacks, against which heretofore the patient had been powerless, except for such small relief as she could get from very hot applications, became less frequent, whilst there was developed much more frequent and severe attacks of frontal pain. Finally the pain in the face was entirely relieved but on the other hand the frontal pain and sense of pressure became permanent. I found rhinoscopically on the side corresponding to the former face pain, viz: the right over the middle turbinated bone, a peculiar tuberculated surface which looked as if it were sown over with numerous small papillomata. The left nasal passage was on the other hand completely normal. I cauterized the right middle turbinated bone thoroughly in two consecutive sittings. She promptly lost the frontal pain and sense of pressure without any return of the pain in the face. Now I believe that this last probably had its origin in contemporaneous nasal affection and could have been cured in the same way, but naturally I am not in a position to prove this assumption.

I will now pass to the discussion of certain single reflex neuroses which seem to me to have had their origin in affections of the pharyngo-nasal cavity.

### SPASM OF THE GLOTTIS.

In this case (10) the patient was a completely healthy physician free from any neuropathic disposition. After he had suffered from night-mare he would be awakened by threatening attacks of spasm of the glottis accompanied by a high grade of dyspnœa. The attacks were short in duration and passed off spontaneously. Posterior rhinoscopy showed considerable injection of the mucous membranes of the naso-pharyngeal cavity, and collection of mucus in this part. Treatment: Pencilling the part with lunar caustic several times repeated. Afterwards he was for a long time free from attacks. They returned, however, with an attack of catarrhal angina from which the patient suffered. Since that there has been no recurrence whatever. This case occurred very opportunely. I had in a former work advanced the hypothesis that reflex spasm of the glottis could perhaps occasionally have its point of irritation in nasal or naso-pharyngeal cavity. At that time I was able only to support my assumption by an experiment in which, with a very sensitive patient, I was able to produce closure of the glottis by touching normal nasal mucous membranes with a sound. The spasm continued several seconds and could only be relieved by a powerful expiration.

### Faucial Cough and Vomiting.

I have selected the following case (11) from a not inconsiderable number of observations, because it most exactly demonstrates the reflex troubles under consideration.

The patient, a physician, complained, though his stomach was most excellent, of a continual tickling cough produced by a constant sensation of having a foreign body in the pharynx, and especially upon rising in the morning he would be attacked with vomiting. On the posterior pharyngeal wall there were a few granulations, but in the clefts behind the tonsils on both sides there were rows of deep red granulations which reached up to the naso-pharyngeal cavity. I cauterized them with the galvano-caustic. The tickling cough ceased during the second sitting, after which all the other troubles passed away.

These important reflexes seem to me to have been too little considered; although C. Mitchel and others have called attention to them. I have had a few cases to treat, in which the patients, on account of the continual vomiting, were thoroughly convinced that they were suffering from stomach trouble and were treated by their physicians accordingly. A single galvano-caustic operation was generally sufficient to remove all the trouble. But even in these cases where there was only coughing and hawking, and this generally only in the mornings, the patients could not express the relief which followed the painless operations so immediately. Here, also, I made the observation that the troublesome symptoms were produced not so much by granulations of the pars oralis—which in numberless individuals produce no inconvenience—as by granulations, which, in all their extent, can only be seen with the aid of the mirror.

I have had occasion to examine in this respect several pregnant women who suffered severely from vomiting. These presented typical granulation rows along the sides of the pharynx. I could not carry out the indicated operative procedure. Whether at a time when, as is well known, the reflexes are generally in a most exalted condition, these troubles may depend upon the increased reflex irritability of these pharyngeal granulations, and whether the hyperemesis of pregnancy can be prevented by a simple opertion more promptly than it can be, according to Friedruch, with bromide of potassium, are questions well worthy of further study. —*Maryland Medical Journal.*

